# Helplessness and perceived pain intensity: relations to cortisol concentrations after electrocutaneous stimulation in healthy young men

**DOI:** 10.1186/1751-0759-5-8

**Published:** 2011-06-30

**Authors:** Matthias J Müller

**Affiliations:** 1 Vitos Clinical Centre Giessen-Marburg Clinic for Psychiatry, Psychosomatic Medicine and Psychotherapy Giessen Academic Hospital, University of Giessen, Licher Straße 106, 35394 Giessen, Germany

## Abstract

**Background:**

Uncontrollable aversive events are associated with feelings of helplessness and cortisol elevation and are suitable as a model of depression. The high comorbidity of depression and pain symptoms and the importance of controllability in both conditions are clinically well-known but empirical studies are scarce. The study investigated the relationship of pain experience, helplessness, and cortisol secretion after controllable vs. uncontrollable electric skin stimulation in healthy male individuals.

**Methods:**

Sixty-four male volunteers were randomly assigned to receive 30 controllable (self-administered) or uncontrollable (experimenter-administered) painful electric skin stimuli. Perceived pain intensity (PPI), subjective helplessness ratings, and salivary cortisol concentrations were assessed. PPI was assessed after stress exposure. For salivary cortisol concentrations and subjective helplessness ratings, areas under the response curve (AUC) were calculated.

**Results:**

After uncontrollable vs. controllable stress exposure significantly higher PPI ratings (P = 0.023), higher subjective helplessness AUC (P < 0.0005) and higher salivary cortisol AUC (P = 0.004, t-tests) were found. Correlation analyses revealed a significant correlation between subjective helplessness AUC and PPI (r = 0.500, P < 0.0005), subjective helplessness AUC and salivary cortisol AUC (r = 0.304, P = 0.015) and between PPI and salivary cortisol AUC (r = 0.298, P = 0.017).

**Conclusions:**

The results confirm the impact of uncontrollability on stress responses in humans; the relationship of PPI with subjective helplessness and salivary cortisol suggests a cognitive-affective sensitization of pain perception, particularly under uncontrollable conditions.

## Background

Uncontrollability of unpleasant life events and aversive stressors seems to be one of the most important determinants of physiological and psychological stress response [1-3]. Learned helplessness theory has shown that repeated exposure to non-contingent feedback, i.e. a lack of correlation between behavior and aversive consequences may lead to negative affective, motivational, and cognitive sequelae including blunted and lowered affect, hopelessness, low self-esteem, motivational deficits and a cognitive bias towards low self-efficacy and controllability expectancies [4,5]. Besides these psychological effects of experiencing uncontrollable stress, activation of the hypothalamic-pituitary-adrenal (HPA) axis, mainly with elevated corticosteroid levels, was repeatedly found after uncontrollable stress [2,6]. Persisting HPA axis activity and hypercortisolism are assumed to be linked to depression and related disorders in humans [7]. On the other hand, depressive and pain-related syndromes are often co-occurring. The comorbidity of depression and chronic pain is very high [8]; other pain syndromes with high prevalence of depressive symptoms comprise fibromyalgia [9] and low back pain [10]. According to clinical and brain imaging studies affective and cognitive factors seem to play a crucial role in modifying and modulating pain experience [11-16]. Cognitions of helplessness, loss of control, rumination and negative future expectations seem to be related to enhanced affective pain experience [9,10,17-20].

Thus, a relationship between helplessness, HPA-axis activation and pain seems to exist in clinical states and disorders, but the findings are controversial. While acute uncontrollable painful stress seems to be regularly followed by a cortisol response [2], in chronic pain syndromes, e.g. in fibromyalgia, blunted cortisol responses and low awakening cortisol levels have been found [21,22]. However, even in patients with chronic pain, affective distress seems to be related to helplessness and enhanced cortisol secretion [23].

Basic psychological stress research in this area is widely lacking. The present study investigated salivary cortisol responses, subjective helplessness, and pain intensity perception (PPI) to controllable and uncontrollable stress in healthy males using an electric skin stimuli procedure. Mildly painful stimuli were used because the main focus of the present study was the PPI in relation to experimentally induced uncontrollability and not the pain induction per se.

It was hypothesized that PPI is intensified and related to salivary cortisol secretion after uncontrollable conditions and experimentally induced subjective helplessness.

## Methods

### Subjects and Design

Healthy male volunteers (age 18-45 years) were recruited by advertisement. After an extensive screening interview individuals with a history of severe medical disease or with a psychiatric disorder or psychotherapy (recently or within the last two years) were excluded. Additionally, volunteers taking any medication potentially interfering with cortisol secretion (e.g. hormones, anti-inflammatory compounds) were excluded. No drinking or eating was allowed at least 2 hours prior to the experiments (4.30 - 7 p.m.). All experiments were carried out at the Department of Psychology, University of Giessen. The present data are part of a larger project comprising also pre-studies, a study with an attention task (one week apart), and several additional assessments not reported here.

Approval by the Institutional Review Board was granted and all subjects had given written informed consent after the procedure had been explained as completely as possible. During the screening session, the electrical stimulus procedure (see below) was explained and individually tested in each participant (1-3 stimuli with the same intensity as used in the study). Sixty-four subjects were randomly assigned to one of the experimental conditions (controllable vs. uncontrollable, see below).

The standardized study protocol comprised baseline (20 min), anticipation (10 min), stress exposure (10 min), and post-stress relaxation (20 min) periods. During baseline conditions the participants were generally informed about the protocol, completed short questionnaires on socio-demographic data and subjective helplessness. In the anticipation period, two silver stimulus electrodes were placed on the non-dominant forearm and fixed with a stretch band, followed by information about the subsequent stress procedure. During the anticipation period, three test trials were carried out.

### Procedure

Mild electric cutaneous stimulation was used to induce completely harmless but potentially painful stimuli according to the literature; the DC electric shock was generated by a transformer/condensor device [24,25]. In a pre-test with 20 healthy students, the lowest intensity which in at least 50% of trials (200/400 trials) was judged at least "mildly painful" (5-point scale of perceived pain: not at all - threshold - mild - moderate - severe) was detected (4.5 points on a scalable potentiometer with an arbitrary intensity scale, 1 - 10). This stimulus intensity (approx. 10 mA) was used in the present trial to assure that all subjects received comparable physical stimulus intensity. All participants were exposed to 30 stimuli with a mean inter-stimulus-interval of about 20-sec (10 min duration of stress exposure).

In each group 32 subjects were investigated. Under "controllable" conditions (C), the subjects could apply the stimulus within an interval of 10 sec at their choice by pressing a button located on the desk. To start a single trial a green LED in front of the participants was activated. If a participant decided not to press the button, the stimulus was automatically applied after 10 sec. In both cases the green changed to a red LED and the stimulus generator was blocked (to avoid more than one stimulus within one interval). A new trial was indicated again by a change of LED activation (from red to green) after the end of the 20-sec interval. Under uncontrollable conditions (UC), the participants stimuli were applied by the experimenter according to a random schedule within the 10 sec interval; all other features of the experiment were identical.

### Assessments

During baseline, anticipation, immediately after the stimuli series, and at the end of the experimental session (relaxation), subjective helplessness was assessed using a previously developed and validated 5-point Likert scale (0-4) consisting of six items ("I feel helpless", "I can (not) influence the situation", " I feel at a loss", "I feel confused", "the situation is inscrutable", "I have (no) control") [25,26]. The scale has good internal consistency (Cronbach's coefficient alpha >0.80). Pain intensity perception (PPI) was judged on a 100-mm visual analog scale (VAS).

Saliva was collected four times for 5 min at the end of baseline (20 min), anticipation (10 min), stress exposure (10 min), and post-stress relaxation (20 min) periods using commercial cotton rolls (Salivette^®^, Sarstedt AG). After centrifugation and saliva specimens were analyzed (double detection). Free cortisol concentrations were detected using commercial sensitive ELISA assays; inter-assay and intra-assay variation was <12%, the lower detection limit was at 1.0 nmol/l.

### Data Analysis

Values are reported as means and standard deviations. For subjective helplessness and salivary cortisol concentrations areas under the response curve (AUC) were calculated according to the trapezoid rule as outlined in the literature [27]. Due to the design and the objective of the present study to sensitively investigate changes in cortisol secretion following a mild stressor in the afternoon, AUCs with respect to increase (AUCi) were calculated [27]. Moreover, negative AUC values could be expected due to the circadian rhythm; in line with the recommendations in the literature, negative AUC values were regarded as "index of decrease" and entered into the statistical analyses [27]. PPI was derived as single assessment after stress exposure. After having tested for normal distribution with Kolmogorov-Smirnov tests (all P-values > 0.15) group differences were analyzed with unpaired t-Tests. Relationships between parameters were evaluated with Pearson correlation coefficients. The level of statistical significance was set at α = 0.05.

## Results

**Figure 1:**
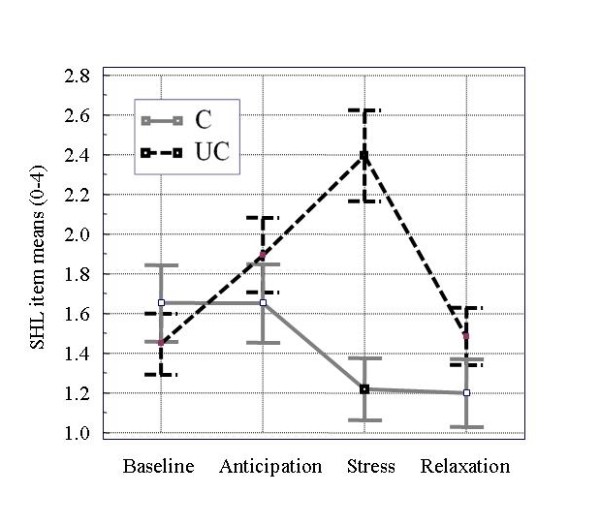
**Subjective helplessness ratings (means ± SEM) under controllable and uncontrollable conditions**. SHL, subjective helplessness; C/UC, controllable/uncontrollable experimental condition

**Figure 2:**
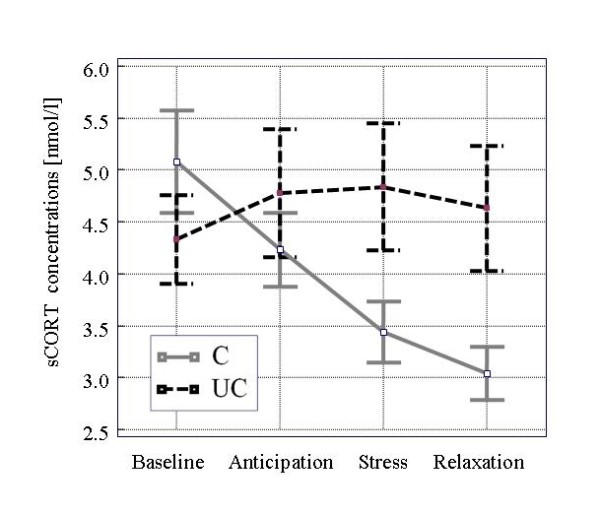
**Salivary cortisol concentrations (means ± SEM) under controllable and uncontrollable conditions**. sCORT, salivary cortisol; C/UC, controllable/uncontrollable experimental condition

The mean age was 25.1 +/- 3.2 years, and 90% of the participants were students. No differences emerged between groups with respect to age, smoking status (52% never smoking, 42% more than 5 cigarettes per day), alcohol consumption (5% never drinking, 59% more than 2 drinks a week), and body mass index (mean 22.3 +/- 1.7 kg/m^2^). Figure 1 and Figure 2 show the course of subjective helplessness ratings and salivary cortisol concentrations under the controllable and uncontrollable conditions.

Baseline subjective helplessness ratings were low and comparable in both groups. Under the uncontrollable stress condition, a sharp increase of subjective helplessness ratings occurred after stress exposure whereas the subjective helplessness ratings values after the controllable condition decreased.

Under baseline conditions, salivary cortisol concentrations were not significantly different (uncontrollable vs. controllable condition). The course of salivary cortisol concentrations in the group under the controllable condition followed strongly the circadian rhythm of cortisol secretion while salivary cortisol concentrations increased slightly in the group with the uncontrollable condition during anticipation and stress exposure.

Table 1 shows the descriptive results in the total group and group comparisons (controllable vs. uncontrollable condition) of PPI, subjective helplessness ratings (AUC), and salivary cortisol concentrations (AUC).

**Table 1:** Results of Perceived Pain Intensity, subjective helplessness, and salivary cortisol levels in the experimental groups

	Total sample (n = 64)	Controllable condition (n = 32)	Uncontrollable condition (n = 32)	t-value (df = 62)	t-test p-value
Pain intensity perception (post exposure)	40 ± 29 (0 - 98)	32 ± 29	48 ± 27	-2.333	P = 0.023
Subjective helplessness AUC (60 min)	8 ± 42 (-135 - 97)	-13 ± 35	28 ± 37	-4.554	P < 0.0005
Salivary cortisol AUC (nmol/l*60 min)	-20 ± 140 (-516 - 452)	-69 ± 124	29 ± 139	-2.979	P = 0.004

AUC, area under the response curve

The AUCs indicate a significantly higher response of cortisol secretion and subjective helplessness after uncontrollable conditions (P < 0.01). The AUC of helplessness ratings was highly correlated with the simple difference of helplessness ratings after stress exposure and baseline (r_ΔSHL;AUC _= 0.93, P < 0.0005).

Mean AUCs of subjective helplessness and salivary cortisol concentrations were negative after controllable stress conditions indicating a decrease compared to baseline. PPI was also significantly more pronounced (P < 0.05) after uncontrollable vs. controllable stress exposure.

**Figure 3:**
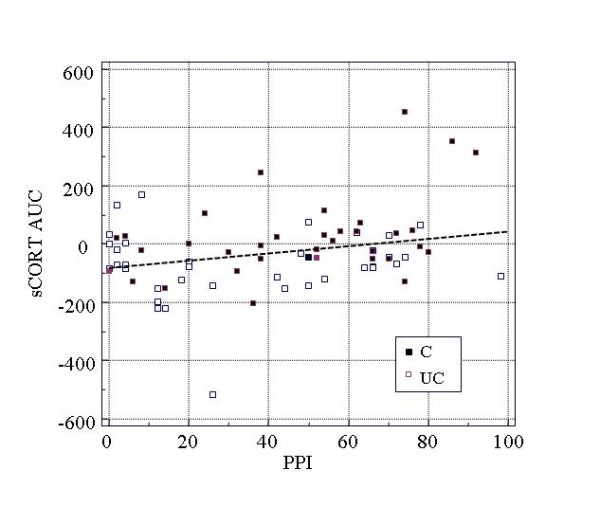
**Correlation of pain intensity perception and subjective helplessness**. SHL AUC, subjective helplessness - area under the response curve; PPI, perceived pain intensity; C/UC, controllable/uncontrollable experimental condition

**Figure 4:**
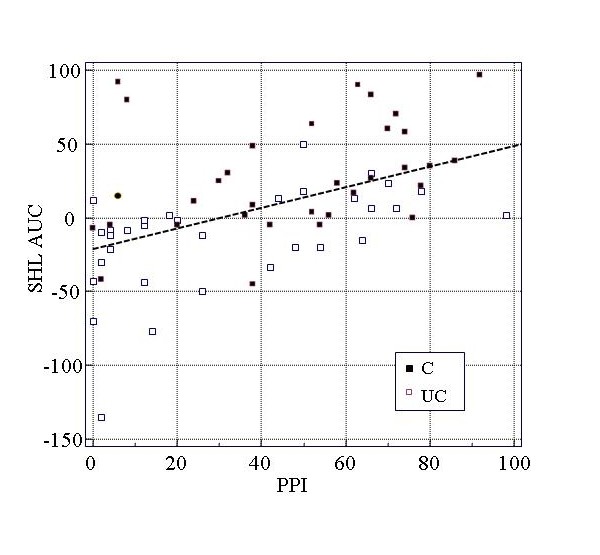
**Correlation of pain intensity perception and cortisol AUC**. sCORT AUC, salivary cortisol concentrations - area under the response curve; PPI, perceived pain intensity; C/UC, controllable/uncontrollable experimental condition

Figures 3 and 4 illustrate the relationships of PPI with helplessness ratings and salivary cortisol concentrations in the total group.

Table 2 reports the correlations between PPI, subjective helplessness, and salivary cortisol in both experimental groups and in the total sample.

**Table 2:** Correlations of Perceived Pain Intensity, subjective helplessness, and salivary cortisol AUCs in the experimental groups

	Subjective helplessness (AUC)			Salivary cortisol (AUC)		
	**Total sample (n = 64)**	**Controllable condition (n = 32)**	**Uncontrollable condition (n = 32)**	**Total sample (n = 64)**	**Controllable condition (n = 32)**	**Uncontrollable condition (n = 32)**

Pain intensity perception (post exposure)	r = 0.500 P < 0.001	r = 0.511 P = 0.003	r = 0.350 P = 0.050	r = 0.304 P = 0.015	r = 0.060 P = 0.745	r = 0.391 P = 0.027
Subjective helplessness AUC (60 min)	-	-	-	r = 0.298 P = 0.017	r = 0.087 P = 0.637	r = 0.202 P = 0.268

Values are Pearson correlation coefficients (r) and corresponding p-values (two-tailed); AUC, area under the response curve

In the total group, significant relationships were found between PPI and subjective helplessness ratings (P < 0.001) as well as salivary cortisol concentrations (P < 0.01) and between subjective helplessness ratings and salivary cortisol concentrations (P < 0.05). Correlations in subgroups (controllable and uncontrollable stress conditions) revealed a significant correlation between PPI and salivary cortisol concentrations (AUC) only in the subgroup with uncontrollable stress exposure. The differences of correlations between the controllable and uncontrollable condition were statistically not significant (P > 0.10).

## Discussion

The main finding of the present study was an association of pain intensity perception with saliva cortisol responses and subjective helplessness after uncontrollable electrical stimuli in healthy young men. After uncontrollable stress exposure, significantly higher pain perception and helplessness ratings as well as a significantly more pronounced salivary cortisol response were found when compared to the controllable stress condition. Moreover, correlation analyses revealed significant positive associations between the three parameters in the total sample without significant differences of correlations between the controllable or uncontrollable condition. Thus, subjective helplessness seems to be a potent cognitive mediator of pain evaluation and HPA-axis activation.

Enhanced pain intensity experience after uncontrollable stress exposure and during states of helplessness is in line with previous findings in healthy subjects and patients with pain syndromes [8,20,28,29]. On the other hand, cortisol elevation following uncontrollable aversive stress has also been a basic finding since the early studies of learned helplessness theory [2,4,30]. However, the relationship between uncontrollable and potentially painful stress, subjective helplessness, and perceived pain intensity has not been sufficiently studied yet. Our results fit closely to very recent data from an interventional study with repetitive transcranial magnetic stimulation (rTMS) [17]. The authors could show that fast left prefrontal rTMS acutely suppressed the analgesic effects of perceived controllability on the emotional dimension, but not on the sensory/discriminatory component of pain perception. After rTMS, perceived uncontrollability of a painful task was related to an emotionally more distressing pain perception; the findings were hypothetically linked to fast activation of left prefrontal cortical areas [17].

The clinical studies in patients with often chronic pain syndromes seem, however, to be contradictory to the present findings. In several studies, lower mean diurnal cortisol levels were found in patients with chronic pain [28,31], particularly with fibromyalgia [22]. After metyrapone-induced hypocortisolism, an increase of mechanical pain sensitivity was found in healthy volunteers [21]. Cortisol response after acute stress in patients with chronic pain seems to be either within the normal range (in patients with chronic pelvic pain) or reduced (in fibromyalgia) [32]. In a recent study of this group [23] diurnal salivary cortisol release was associated with depression in patients with fibromyalgia, but not with perceived pain. Another recent study investigated the impact of perceived control during a cold pressor test and the influence of active coping on salivary cortisol response and reported a weak interaction of high perceived control and active coping on higher cortisol responses which occurred only in women [33]. In men, a reverse picture emerged. The authors claim that cortisol elevations after acute painful stress could be an adaptive neuroendocrine mechanism and interpreted their result as evidence that active coping and perceived control could potentiate adaptation [33]. Although an adaptive function of cortisol responses after acute uncontrollable painful stress can not be ruled out, converging evidence shows, however, that negative cognitive and affective factors intensify both HPA axis activation and pain perception. Anticipatory and evaluative cognitions seem to be crucial for pain processing [8,15,34] and cortisol response [28,35]. Most likely blunted HPA axis reactivity and hypocortisolism as seen in post-traumatic stress disorder and fibromyalgia are consequences of chronic stress and a prolonged period of HPA axis hyperactivity [36]. Our study suggests that acute painful stimulation is not followed by HPA axis activation under controllable conditions and when the perceived level of helplessness is low. Under such conditions pain was perceived less severe compared to uncontrollable stress exposure and states of induced helplessness.

However, generalization of our findings should be limited to healthy young men. Gender differences in stress response and pain perception should be taken into account [31,33]. An influencing factor which has not been ruled in the present study was tobacco smoking. Smoking can activate the HPA axis, but non-smokers and smokers were equally distributed in both experimental groups.

Salivary cortisol responses were relatively small due to the mild stimulation compared to other stressors [37]; the pain stimulation procedure used in the present study was quite artificial and might have led to a stimulation of both non-nociceptive and nociceptive fibers. Additionally, stress induction and measurement of altered pain intensity were implemented concurrently. Stressor modality, intensity and the temporal pattern of stress exposure seem all to have influence on pain processing [38] and cortisol responses. The present findings are, therefore, in need for replication.

## Conclusions

The study presents experimental data of healthy males corroborating the hypothesis that perceived controllability of painful stimuli is crucial for perceived pain intensity and HPA axis activation. The findings can help clinicians substantiate and foster cognitive-psychotherapeutic approaches to prevent and treat helplessness in the context of pain management.

## Abbreviations

AUC: area under the response curve; C/UC: controllable/uncontrollable conditions; HPA axis: hypothalamic-pituitary-adrenal axis; PPI: perceived pain intensity; sCORT: salivary cortisol; SHL: subjective helplessness; VAS: visual analog scale.

## Competing interests

The author declares that they have no competing interests.
